# External Use of Chloral Hydrate

**Published:** 1875-09

**Authors:** 


External Use of Chloral Hydrate.—A writer in the *Canada Medical Record* recommends a solution of chloral hydrate in water, from three to five grains to the ounce, as a substitute for carbolic acid for external use. Its effect on ulcers is prompt and admirable, and a case of eczema was successfully treated by it. In cases of amputation, where the surfaces took on unhealthy action, the solution changed their character and removed the difficulty in twenty-four hours. It removes fetor, is clean and neat, does not stain, and stimulates granulations without destroying them.

Chloral hydrate has also been used in Germany as an antiseptic application in cancer of the uterus. A strong solution—one part to twelve of water—is applied by means of cotton. Dr. Goodell of Philadelphia also speaks highly of it as a purifying and deodorizing agent.—*Pacific Med. and Surg. Jour.*

The *Medical and Surgical Reporter* says: "Framing diplomas for one's office is entirely proper. The card you send announcing 'calls promptly attended to, day or night, city or country,' is not contrary to ethics, but appears to us in questionable taste."

Prof. Pajot, of Paris, sums up his views upon anæsthesia in labor thus: "*True anæsthesia* applied to labor is a serious and scientific proceeding that can be discussed. Its dangers and disadvantages appear to us greater than its advantages.

The pretended *semi-anæsthesia* is as useless as inoffensive. It has nothing scientific or serious about it. It can be placed alongside those other dilatory means that act on the imagination of the woman, and that gain time, when in natural labor there is need of nothing else. They can make women think they would have suffered more had it not been for the semi-anæsthesia. Chloroform will be for the woman in child-bed like Providence, that it is always necessary to thank when one breaks one leg—both might have been broken.—*Med. Exam.*

"A Reseat fore the Sistum."—The following "Reseat" was given by a traveling doctor to an Indiana man, who paid him ten dollars for it and his advice:

Sasaprile theard of . . . . . . 0℥
Wahoon theard of . . . . . . . 0℥
Wild Cherry quartr of . . . . . 0℥
One pint of Alkhol.
2 pound of Shugr.
One pint of wattr.
Conmlit All.
pr dos one swaller 3 times pr dy Before Eating.

